# An Essay on the Treatment of Compound and Complicated Fractures

**Published:** 1846-07

**Authors:** 


I. An Essay on the Treatment of Compound and Complicated Fractures. By *W. T. Walker*, M.D. Octavo, pp, 100. Boston, U. S. 1845.

II. De l'Amputation des Membres par Suite des Plaies d'Armes a feu. Par *J. Lisfranc*. (Bulletin General de Therapeutique, 1845.)

On Amputation in Gun-shot Wounds. By *J. Lisfranc*.

The object of Dr. Walker's Essay is to prove "the powers of Nature in healing compound fractures attended with great contusion, laceration, and loss of substance of the bones, and of the skin, and other soft parts, to be much greater than is generally supposed." To this end he relates six cases which fell under his own notice, and quotes several others from the older surgical writers, whose precepts, he considers, have not been sufficiently attended to in modern times.

"The precepts to which I allude are—1. That all tendinous or membranous structures which obstruct the removal of foreign bodies, or unduly confine or strangulate the soft parts, when swollen by inflammation, should be promptly and freely divided. 2. That such dependent orifices should be preserved, or counter-openings made, as will, when aided by position and dressings, secure the free discharge of all fluids which might otherwise stagnate within the wound. 3. That portions of bone protruding through the integuments, which cannot, easily and without violence, be reduced to their proper place, should at once be removed by the saw; and that all foreign bodies, loose portions or shivers of bone, should be promptly extracted from the wound. 4. That great pain, inflammation, or nervous symptoms, depend rather on peculiar complication than on the extent of the wound. And that they indicate great danger unless rightly understood and appropriately treated and relieved."

The recognition of the vast extent of Nature's restorative powers is one of the just boasts of our actual surgery, and has caused a great diminution of that hasty lopping-off of limbs which not long since disgraced its votaries, both in this country and on the Continent. That this greater caution extends to cases involving bad fractures of the extremities is familiar to all conversant with the deliberation with which our hospital surgeons now weigh the possibilities of being able to dispense with an operation, justly deeming this one of the greatest triumphs of their art; and, indeed, as regards our own country, we are prepared to maintain that the whole history of surgery does not supply a period so characterized by an enlightened abstinence from officious intermeddling with Nature's work. The cases culled by Dr. Walker from the practice of Paré, Wiseman, De la Motte, and others, may easily find their parallels in that of many of our surgeons; they are indeed exceptive, and as a general rule amputation was far more frequently resorted to in such accidents at the epoch when these celebrated men lived than it is at present. However we do not mean to state that we have yet reached the attainable limits, but it is quite certain there is a disposition to extend these as far as possible; and Mr. Rutherford Alcock's papers* so recently published have shewn us that a class of cases, bad wounds of joints, hitherto excluded, may oftentimes be placed in the category. It is, however, no such simple matter to determine upon the propriety of non-operating, as it would seem; for we have to decide not only that there is strong probability of life being saved, but also that an effective limb will be preserved. The constitution, condition of life, and means of effectually treating the patient, have to be considered quite as attentively as the nature and extent of injury he has undergone; for it would be but sorry humanity to attempt to save the limb of one whose powers were incompetent to the necessary restorative processes, or when it would never prove other than a painful incumbrance and impediment.

An important principle laid down by Dr. Walker is, that whatever interference the injured extremity may require should be resorted to at once, rather than delayed until the inflammatory process is set up. Indeed, where amputation itself is demanded, the author agrees with the great majority of surgeons of the present day, that it should be performed *immediately*, or at least within the first twenty-four hours. This too was the generally received doctrine in the profession until the French Academy of Surgery awarded its medal in 1754 to M. Faure, an army surgeon, who maintained that the operation should be deferred until after the establishment of suppuration. He stated, that of more than 300 persons operated upon immediately after the battle of Fontenoy thirty only escaped, while of 10 operated upon after the subsidence of the first symptoms, all recovered. Dr. Walker believes the opinions of Hunter upon this point, which were published some time afterwards, and which exerted so injurious an effect upon practice in this country, were based upon the authority of Faure. The experience of the last war completely exploded them as regarded military practice; but they long retained a hold upon practitioners in civil life. The fallacy of Faure's arguments were indeed exposed, but unsuccessfully, by M. Boucher, a cotemporary shortly after the award of the Academy had been made. He shewed that Faure had reasoned from cases in which the performance of the operation had been undertaken after inflammatory action and fever were set up, whereas the true object of immediate amputation is to anticipate these. Moreover, it seems there was great lack of surgeons at Fontenoy, so that few of the sick could receive due care. Dr. Walker thus extends the application of the above remark.

"If this be the explanation, and I doubt not that it is, then it is established, that in the days of Faure and of Hunter, as well as in our own, neither amputation, nor other operation of surgery, can be performed upon the human body while suffering under inflammation and fever, without incurring the greatest danger. * * * * If, then, amputation is not to be performed in a compound fracture, from the commencement of the second day after the injury until suppuration has been established, does it not follow, by a parity of reasoning, that we should avoid, during the same period, all acts which may promote or aggravate inflammation? Does it not also follow, that every thing possible

* Med.-chir. Trans. vol. 23.

should be performed on the first day, and that every act which can, in any way, excite or aggravate inflammation or irritation should be studiously avoided during the intervening time preceding suppuration? Is it not clear that all membranous structures, which, in consequence of the injury, have become so many bands, impeding or even arresting the circulation of blood, or the transmission of nervous influence, should be at once divided? Shall we, in compliance with the rules of our art, remove from our bandages both hem and seam, lest they irritate or cause ulceration of the skin, and at the same time suffer rough, angular, loose fragments of bone or other foreign bodies to remain within wounds to irritate, inflame, and endanger the vital structures they may lie in contact with? Shall we any longer talk about the necessity of cleanliness, and busy ourselves with water, soap, and sponge, in washing the external coverings of a broken limb, knowing full well at the time that this very limb is, within, full of all uncleanliness? Shall we recognize any longer as a principle of our art, that in such cases, it is Nature alone which has power, after weeks of suffering and danger, to relieve our patients, by removing the complication of their wounds, whence all their sufferings and danger come?"

Dr. Walker combats the opinion of Sir James Earle, that the serious symptoms of compound fracture are dependent upon the admission of air, an opinion founded upon the apparent analogy of the hectic and other fatal symptoms which supervene upon the opening of psoas abscesses, &c.

" These views are certainly not sustained by the cases collected and hereto annexed, as neither hectic, inflammation of a grave character, or severe pain. or nervous symptoms have occurred in either of them, where the wound was at once sufficiently open, drained of liquid discharges, and freed from all foreign bodies; while severe symptoms have attended some of the other cases, where these precautions have not been sufficiently regarded; yet here, on several occasions, the severe symptoms vanished as soon as the needful treatment was adopted. I will only add that I have, on many occasions, known compound fractures heal readily as by first intention; but this result has only been obtained in cases where the wound has been inflicted on the bones and muscles, without splintering or other important complication; while the severest symptoms have followed such fractures as have occurred in tendinous or membranous parts, where the bone was much splintered, and the skin but little broken. Most English and American surgeons, however, have adopted the views of Sir James Earle, and have endeavoured to convert compound into simple fractures, by closing the wound by means of adhesive plaister and bandage, in the hope of healing by the first intention. Now, although there may be but little objection to gently drawing the lips of the wound together by adhesive plaister, in the simpler kinds of compound fracture, I mean such as are compound by definition, not by complication, yet I apprehend much mischief may flow from the practice, if extended to the more complicated forms of fractures, especially when, in addition, graduated compresses and bandages are made use of to prevent the undue rising of the bone. Under such treatment I doubt not that inflammation and sloughing have often been attributed to the original injury, which would never have existed, had it not been for undue constriction over the wound."

The only other point we have to notice in Dr. Walker's interesting Essay is the recommendation of early *dilatation of the wound for the extraction of foreign bodies.* All the celebrated older surgeons, such as Paré, Wiseman, Petit, Ledran, &c. recommended that, where the case is such as to require this procedure, it should be put into force at once. They maintained that dangerous symptoms arose, unless tension was removed and foreign bodies, &c. were extracted prior to the setting up of inflammation. This was quite consistent with their opinion of the preferableness of prompt to deferred amputation. Hunter, Pott, Abernethy, and most other surgeons, however, disapprove of considerable attempts at the removal of foreign bodies being made prior to the occurrence of suppuration, and offer no recommendation of early dilatation of the wound for draining it of its fluids or for the relief of tension ; and even Mr. Guthrie,* the able advocate for early amputation, recommends delaying dilatation and the removal of fragments of bone, &c. until suppuration is established. This advice Dr. Walker considers both inconsistent and injudicious, and much prefers that delivered by the older authorities referred to. We believe, indeed, the tedious progress of many of these cases might be prevented by a somewhat more active and earlier interference than most modern surgeons seem disposed to employ; but, on the other hand, great additional irritation would necessarily be excited, and additional suffering caused, if that excessive probing and searching for foreign bodies put into force by the old surgeons were revived.

Connected with this subject, we are desirous of furnishing an abstract of a paper upon *Amputation in Gun-shot Wounds,* recently published by M. Lisfranc. It is gratifying to find a surgeon of his eminence, and operating celebrity, expressing such *conservative* views upon this point, albeit he may hold others *destructive* enough upon some other subjects in surgery. Before adverting to those cases in which the bones are fractured, he observes that, when about two thirds of the *muscular substance* at the upper portion of the thigh have been torn off by a ball, amputation should be resorted to, even although no very important vessels or nerves be injured. Cicatrization in such a case becomes tedious and difficult; and the cicatrix which is formed is thick, adherent, large, and easily torn ; while the movements of the part after so large a loss of muscular substance become impaired or impossible. The dangers and unsatisfactory results of such cases always lead to regret when the operation has been declined. A similar wound, occupying the arm, by no means renders an operation so essential.

Some modern works have stated that wounds of *joints* are least dangerous when the ball only makes two small apertures while traversing them. So far is this from being the case, however, owing to the absence of due exit for the fluids which result from the accident, that M. Lisfranc has found the happiest effects follow the enlargement of the wound, as recommended by Larrey ; and, providing the loss of substance be not very great, there is therefore less danger in a large than a narrow wound. " I have shewn, in a great number of cases, that where the loss of substance is not very large, and appropriate after-treatment is pursued, bad wounds of the shoulder, elbow, wrist, knee, or ankle do not necessitate amputation." He makes the following statements respecting fractures.

" It is generally believed that amputation is required when the body of a long bone of the lower extremity is fractured in a comminuted manner. I reject this precept. Lombard, Gaultier de Claubry, S. Cooper, Percy, Larrey and Guthrie, all state they have seen but few such cases of fracture of the femur recover, almost all dying. Ribes did not obtain one cure out of 10 patients, upon whom

---

* Med.-Chir. Review, April 1838, (Appendix).

every care was bestowed; and among the 4000 old soldiers at the Invalides, he could not find one who had been thus cured of this accident; but it is to be observed these latter cases were treated in the army, where sufficient care cannot always be furnished or due transport obtained. It is said no better success has attended these cases in the Belgian and Paris Hospitals, but I suspect this arises from appropriate measures for the prevention and removal of inflammation not having been put into force. I believe, as a general rule, we should not resort to amputation in these cases before inflammation has declared itself and resisted an appropriate plan of treatment. This, independently of phlebitis and resorption, is all we have to fear in comminuted fracture. I must, however, admit that my opinion is as yet scarcely based upon a sufficient number of facts, and that I have chiefly founded it upon the analogy of what takes place in the upper extremities, in which comminuted fractures produced by fire-arms hardly ever give rise to fatal accidents. Dupuytren, however, cured a case of bad fracture of the femur. I have saved several, and not yet lost one. In 1830, I had two patients brought in, both of whom did well. In the first, there was a comminuted fracture of the femur, and in the other, there was great accompanying crushing of the soft parts. In both cases the *constitution was good and the intestinal canal in excellent condition*—important points for consideration in deciding whether amputation can be dispensed with.

"It has been said that such cases, even when cured, are almost always followed by fistulæ and *engorgements*, which impair the usefulness of the limb, and eventually induce death in a great number of instances. I do not admit this. I have retained my army connexions with certain officers, in whom such fistulous openings manifested themselves. It is true that swellings arising from the presence of pus are formed from time to time, which disappear when it is evacuated. These persons walk pretty well, are but little annoyed at the slight discharge which persists, and are in a far better state than if they had undergone amputation, which is not, be it recollected, unfrequently a fatal operation. The fistulæ may be complicated with considerable *engorgement*, of the limb. Sometimes these are the results of the primary inflammation, and at other times they arise from neglected or badly treated phlegmasiæ, produced by the presence of pus in the sinuses. In this latter case, relief is obtained when the pus is evacuated; and in all cases powerful means of combatting simple induration have been long known, such as careful local bleedings when there are phlegmasiæ, and resolvents when these are absent.

"Fractures complicated with solution of continuity of the soft parts, and produced by ordinary causes, are less dangerous than those resulting from fire-arms; and are also by far less so when affecting the upper than lower extremities. It is sometimes difficult enough to distinguish the circumstances under which the amputation should be determined upon or rejected. If the locality is a good one, the patient's constitution and digestive canal in good order, if his *morale* is good and his animal economy unaffected by any cachexia, we need rarely operate; for under these circumstances, success frequently surpasses all expectation. I am fond of saying to the young pupils who come to study in the hospitals of large cities, that they must distrust the observations they make there, and recollect that, under other circumstances, the removal of limbs should be much oftener rejected.

"When the principal vessels of the limb are divided, large ecchymoses or effusions of blood are present, the soft parts are largely torn or much bruised, or a large joint is opened, we must amputate; but we need not if the vessels have been saved, or the joint is not open to the air. When the fracture is comminutive, the effusion of blood large, and the muscles torn off to some extent, I have frequently applied my usual measures at La Pitié, and every one is aware of my success in saving these limbs, several of which had been crushed by the wheels of carriages. When the fragments have traversed the skin, and we are unable to reduce them owing to the narrowness of the aperture, we should enlarge the incision, and remove any portion of bone which is denuded of periosteum.

"There is much difference of opinion among surgeons as to the propriety of amputation in these cases. I believe opinions would be much less divergent, and even all contest terminate, if medical knowledge as well as surgical were more generally diffused, and if preconceived and exaggerated ideas acquired at bad schools, did not so frequently serve as a guide to the practitioner. Read the accounts of cases that have been published, and you will find in the majority of these that true medical ideas have been completely neglected."

Throughout his paper M. Lisfranc frequently reiterates that, if limbs are to be saved from amputation, under the circumstances mentioned above, the patient must be treated upon the plan he has repeatedly made public.* This consists in the observance of a rigid diet, the employment of several small bleedings from the arm (discontinuing these when suppuration is about to occur), and the frequent application of emollient cataplasms to the parts. We may observe also, that Mr. R. Alcock, in cases of wounds of the joints which he wishes to save from amputation, has recourse to active general and local bleeding, and the application of slightly stimulating fluids. When we consider that these accidents so frequently occur to persons in robust health, we certainly think that enough use of depletion is not, as a general rule, made by our surgeons, who, indeed, seldom resort to it at all.

* See Med.-Chir. Review, Jan. 1843, p. 63.

